# Mechanism of AMPK-mediated apoptosis of rat gastric smooth muscle cells under high glucose condition

**DOI:** 10.1042/BSR20192504

**Published:** 2019-12-13

**Authors:** Xiang-zi Zhang, Mo-han Zhang, Xue-sen Fang, Xiang-shu Cui, Zheng Jin

**Affiliations:** 1 Department of Histology & Embryology, Yanbian University College of Medicine, Yanji 133000, China; 2 Department of Humanistic Nursing, Yanbian University College of Nursing, Yanji 133000, China

**Keywords:** AMPK, cell apoptosis, diabetic gastroparesis, high glucose, rat gastric smooth muscle cells

## Abstract

To observe changes in AMP-activated protein kinase (AMPK) activity and phosphorylation changes in AMPK signaling pathway in gastric smooth muscle cells of rats with diabetic gastroparesis (DGP), investigate the effect of AMPK on apoptosis and explore the underlying mechanism. After establishing rat model of DGP, rats were divided into normal control (NC) and DGP groups. The phosphorylation changes in AMPK pathway were detected by AMPK Signaling Phospho-Antibody Array, and the apoptosis-related proteins were determined. Rat gastric smooth muscle cells were cultured *in vitro* under different glucose conditions, and divided into normal and high glucose groups. The AMPK activity and intracellular Ca^2+^ changes in cells were observed. After AMPK silencing, cells were divided into high glucose-24h, high glucose-48h and high glucose-48h+siRNA groups. Changes in expression of apoptosis-related proteins were observed. AMPK activity and apoptosis rates were both increased in gastric smooth muscle tissues in DGP rats (*P*<0.05, *P*<0.001, respectively). A total of 14 apoptosis-related differentially phosphorylated proteins were identified. Under high-glucose condition, AMPK activity and intracellular Ca^2+^ concentrations in rat gastric smooth muscle cells were increased (both *P*<0.05). After AMPK silencing, p53 expression was decreased, Akt and p70 S6 ribosomal protein kinase (p70S6K) activities were were increased, Bcl-2 expression was increased, CaMKII activity was decreased in the high glucose-48h group. Under high-glucose condition, activated AMPK can directly or indirectly promote cells apoptosis by regulating the expression and activity of p53, Akt, p70S6K, Protein kinase A (PKA), Phospholipidol C (PLC)-β3, CaMKII, CaMKIV and eukaryotic translation initiation factor 4E binding protein1 (4E-BP1) in rat gastric smooth muscle cells.

## Introduction

Diabetic gastroparesis (DGP) is one of the common complications of diabetes, which is characterized by decreased gastric motility-induced delayed gastric emptying. Although gastric smooth muscle motility is regulated by nerve and body fluids, however the contribution of the functional status of gastric smooth muscle cells in regulating smooth muscle motility cannot be neglected. The contractile activity of gastric smooth muscle is the power source of gastric emptying, and the effective gastric smooth muscle cell numbers and sufficient energy supply are critical for gastric smooth muscle contraction. Finding certain factors affecting gastric smooth muscle cell apoptosis and determining the relevant mechanisms have a positive role in the treatment of DGP and even diabetes.

AMP-activated protein kinase (AMPK) is expressed in various tissues and organs and can be activated by multiple stimuli. AMPK plays an important role in regulation of many physiological and pathological processes in cells and is considered an important target for treatment of diabetes and other metabolic diseases. Our previous study showed that AMPK activity in rat gastric smooth muscle tissues was decreased first and then increased during the development of DGP, indicating that AMPK activity is involved in the occurrence of DGP [1]. AMPK is a heterotrimeric protein composed of a catalytic α subunit and two regulatory subunits, β and γ [2]. AMPK activity is regulated by phosphorylation on Thr^172^ located on the α catalytic subunit. Studies have found that AMPK is involved in apoptosis by affecting reactive oxygen species (ROS), mammalian target of rapamycin (mTOR), Bim, and p53 pathways [2,3]. However, the exact mechanism of its effects on apoptosis under high glucose conditions is still unclear.

In the current study, we established a DGP rat model, cultured rat gastric smooth muscle cells *in vitro*, observed changes in AMPK activity and the phosphorylation changes in AMPK signal pathway, investigated the effect of AMPK activation on apoptosis, and elucidated its underlying mechanisms. The study aimed to clarify the pathogenesis of DGP and provide experimental evidences for the treatment of GDP.

## Materials and methods

### Materials

Twelve adult male Sprague–Dawley (SD) rats, weighing 200 ± 20 g, were provided by the Experimental Animal Center of Yanbian University. Rat gastric smooth muscle cells (iCell Bioscience Inc, Shanghai, China), streptozotocin (STZ, Cat No. S0130, Sigma, MO, U.S.A.), phospho-AMPK antibody (Cat No. ab133448; Abcam, Cambridge, U.K.), AMPK antibody (Cat No. ab131512; Abcam, Cambridge, U.K.), internal reference β-actin (A5316, Sigma, MO, U.S.A.); Annexin V-FITC/PI apoptosis detection kit (Cat No. 556547, BD Biosciences, Heidelberg, Germany), AMPK Signaling Phosphorylation Antibody Array (Cat No. PAM174, Full Moon BioSystem, CA, U.S.A.); p53 antibody (Cat No. 32532, Cell Signaling Technology, Inc. MA, U.S.A.), Bcl-2 (Cat No. 196495, Abcam, Cambridge, U.K.), BAX (Cat No. ab182734, Abcam, Cambridge, U.K.), Caspase-3 (Cat No. ab90437, Abcam, Cambridge, U.K.), Akt antibody (Cat No. 9272, Cell Signaling Technology, Inc. MA, U.S.A.), Phospho-AktSer^473^ antibody (Cat No. 4060, Cell Signaling Technology, Inc. MA, U.S.A.), p70 S6 ribosomal protein kinase (p70S6K) antibody (Cat No. 9202, Cell Signaling Technology, Inc. MA, U.S.A.), Phospho-p70S6K Thr^389^ (Cat No. 9205, Cell Signaling Technology, Inc. MA, U.S.A.), eukaryotic translation initiation factor 4E binding protein1 (4E-BP1) antibody (Cat No. 9452, Cell Signaling Technology, Inc., MA, U.S.A.), CaMKII antibody (Cat No. ab52476, Abcam, Cambridge, U.K.), Phospholipidol C (PLC)-β3 antibody (Cat No. 14247, Cell Signaling Technology, Inc. MA, U.S.A.), Phospho-CaMKII Thr^305^ antibody (Cat No. SAB4503756, Sigma, MO, U.S.A.), CaMKIV antibody (Cat No. ab3557, Abcam, Cambridge, U.K.), Phospho-CaMKIV Thr^196/200^ antibody (Cat No. ab59424, Abcam, Cambridge, U.K.), PKA Kinase Activity Assay Kit (Cat No. ab139435, Abcam, Cambridge, U.K.), AMPKα1/α2 siRNA (production by RiboBio Company, Guangzhou, China), AMPKα antibody (Cat No. 2532, Cell Signaling Technology, Inc. MA, U.S.A.), 2-Deoxy-d-glucose (2-DG, Cat No. S4701, Selleck Chemicals LLC, TX, U.S.A.), α-SMA (Cat No. ab32575, Abcam, Cambridge, U.K.).

### Diabetic rat model establishment and grouping

A total of 0.5% of STZ solution was prepared in 0.1 mol/l citrate buffer. Rats were housed individually in single cages at a relative humidity of 50–80%, and room temperature of 18–25°C with 12-h light/dark. And rats were allowed free access to food, water, and adapted to the laboratory conditions for 1 week. Then they were fasted for 12 h with free access to drinking water. After weighing, they received a single intraperitoneal injection of STZ (65 mg/kg), and housed under same conditions. After 7 days of injection, tail vein blood was taken, and the blood glucose concentration > 350 mg/dl indicated successful establishment of diabetic rat model. According to the results from our previous study, DGP was developed at 8 weeks after diabetic rat model establishment (DM8w) [1]. Rats were randomly divided into DGP and normal control (NC) groups with ten rats in each group. The present study was carried out in the Laboratory Animal Center of Yanbian University College of Medicine in strict accordance with the recommendations in the Guide for the Care and Use of Laboratory Animals. All animal experimental procedures were approved by the Ethics Committee of Yanbian University College of Medicine.

### Detection of gastric smooth muscle cell apoptosis of DGP rats by flow cytometry

According to the method of our previous study [1], rats were adapted to the laboratory conditions for 1 week and fasted for 12 h with free access to drinking water, all rats were killed by cervical dislocation. The gastric smooth muscle tissues were isolated, cut into fine pieces with scissors and filtered. After centrifugation at 1500 rpm for 4 min, the supernatant was discarded and the pellet was re-suspended in DMEM. Then times volume of 0.1% collagenase was then added and stirred in a water bath at 37°C. Cells were collected every 30 min for a total of three times, and centrifuged for 3 min at 1500 rpm each time. After discarding the supernatant, cells were resuspended in DMEM and mixed to generate a cell suspension. Necrotic cells were discounted by Trypan Blue staining. α-SMA expression was observed by immunofluorescence assay, and smooth muscle cells were identified. Cells were counted, and cell density was adjusted to 1 × 10^6^ cells/ml. Cells were washed twice with PBS, centrifuged at 2000 rpm for 5 min, and 1 × 10^5^ cells were collected and resuspended in 500 μl binding buffer. After 5 μl Annexin V-EGFP was added and mixed, 10 μl PI was added and mixed. This mixture was incubated in the dark on ice at room temperature for 5–15 min. Flow cytometry was used to observe apoptosis of cells in each group.

### AMPK Signaling Phospho-Antibody Array

Six rats in NC and DGP groups were selected. AMPK Signaling Phospho-Antibody Array was perfomed in two groups. Results was scanned on Microarray scanner and analyzed, the samples can also be stored in the dark at −20°C and scanned within 3 days. The expression levels of apoptosis-related proteins were determined.

### Detection of AMPK activity in rat gastric smooth muscle cells cultured under high glucose condition by Western blot analysis

Rat gastric smooth muscle cell in the logarithmic growth phase were obtained, and divided into normal glucose (5 mmol/l) group, high glucose (35 mmol/l) group. At 24 and 48 h, cells in the logarithmic growth phase were obtained from each group, medium was discarded and replaced with PBS. Total proteins were exacted from cells, and the protein concentration of samples was determined by a full-wavelength spectrophotometer. Samples were boiled for 3 min and 40 μg of protein were loaded into each well of a sodium dodecyl sulfate/polyacrylamide gel (SDS/PAGE, 10%). After electrophoresis separation, proteins were transferred on to PVDF membranes using the semi-dry transfer method. Membranes were blocked with 5% skimmed milk powder in TBS-T buffer solution (25 mmol/l Tris, 150 mmol/l NaCl, 1% Tween 20, pH 7.5). After washing, blocked membranes were incubated with antibodies against phospho-AMPK (1:1000), AMPK (1:1000), β-actin (1:500) at 4°C overnight. After washing, membranes were incubated with a horseradish peroxidase (HRP)–conjugated goat anti-rabbit IgG secondary antibody (1:1000) at room temperature for 1 h. Finally, membranes were washed and exposed with a gel imaging analysis system. Images were analyzed and the phospho-AMPK/AMPK ratio was calculated.

### Confocal laser scanning microscopy detection

Primary cultured rat gastric smooth muscle cells were divided into normal glucose (5 mmol/l) group, high glucose (35 mmol/l) group, confocal laser scanning microscopy detection was performed at 24 and 48 h of culture. When cultured cells reached 80% confluence, Fluo-4 AM was diluted to 0.5–5 μM in PBS. After removing the culture medium, the cells were washed three times with PBS or HBS, then fluo-4 AM working solution were added in an amount sufficient to cover the cells, after incubation at 20–37°C for 10–60 min, the cells were washed three times with PBS or HBSS, then cells were reincubated with fluo-4 AM working solution for another 20–30 min to ensure that fluo-4 AM was converted into its functional form fluo-4 in the cells. If necessary, the cells can be stimulated by appropriate drugs. The fluorescence of fluo-4 was detected using confocal laser-scanning microscopy to determine the changes in intracellular Ca^2+^ concentration. It should be noted that apparatus should be kept away from potential sources of electromagnetic radiation, the room for detection should be free from vibration and shocks. To make sure samples are not bleached with light, the room should be darkened, clean, range in temperature from 5 to 25°C.

### Silencing of AMPK with RNA interference

Rat gastric smooth muscle cell in the logarithmic growth phase were obtained, cell suspensions were prepared and added into a six-well plate at a cell density of 10^5^ cells per well in 2-ml culturing media, and then cultured at 37°C overnight. Electroporation was performed when cultured cells reached 80% confluence. A 0.1 cm electroporation cuvette was pre-chilled on ice for 5 min, 1 μl of purified ligation product or 0.5 μl of unpurified ligation production was added to 100 μl cells, mixed well and placed on ice for 20 min. The mixture was added into the pre-cooled electroporation cuvette (attention should be paid to keep the outside of the electroporation cuvette dry and to avoid sparks), then the cuvette was placed in the holder in the electroporation apparatus and shocked in a Bio-Rad Gene Pulser at 3 kV with a 25 µF capacitor and 200 Ω parallel resistor, and with time constant of 4.5–5 ms. After a buzzing sound is heard, 1 ml SOC resuscitation medium (37°C) was added to the cuvette quickly, after washout of the mixture, the mixture was transferred to a 1.5 ml centrifuge tube, and incubated at 37°C in a shaking incubator (220–250 rpm) for 1 h. Afterward, cells were plated on plates, plates were then inverted in an incubator at 37°C for 24–48 h incubation, and the cells were collected for subsequent experiments.

### Detection of apoptosis-related protein expression in rat gastric smooth muscle cells cultured under high-glucose condition after AMPK silencing by Western blot analysis

Rat gastric smooth muscle cell in the logarithmic growth phase were obtained, and divided into high glucose (35 mmol/l)-48h group, high glucose-48h+siRNA group and high glucose-24h group (control gorup). The procedures were the same as those mentioned above. Antibodies were p53 (1:1000), Akt (1:1000), Phospho-AktSer^473^ (1:1000), p70S6K (1:1000), Phospho-p70S6KThr^389^ (1:1000), 4E-BP1 (1:1000), CaMKII (1:1000), Phospho-CaMKII Thr^305^ (1:1000), PLC-β3 (1:1000), CaMKIV (1:1000), Phospho-CaMKIV Thr^196/200^ (1:1000), Bcl-2 (1:1000), BAX (1:1000), Caspase-3 (1:1000), β-actin (1:500).

### Detection of protein kinase A activity in rat gastric smooth muscle cells cultured under high-glucose condition after AMPK silencing by Enzyme-linked immunosorbent assay

Rat gastric smooth muscle cell in the logarithmic growth phase were obtained, and divided into high glucose-48h, high glucose-48h+siRNA and high glucose-24 h groups (control group). All prepared reagent solutions (wash solution, biotin–conjugated antibody working solution, and HRP–conjugated avidin working solution) were equilibrated to room temperature (18–25°C) for at least 30 min before use. Standard and sample wells were set, 100 μl of the standards and samples were added into each well, respectively, the solutions in all wells were well mixed by shaking the plate gently, then the plate was sealed with a plate cover, and incubated at 30°C for 2 h. After discarding the liquids and drying the plate without washing, 100 μl of biotin–conjugated antibody working solutions were added to each well, then the plate was sealed with a plate cover, and incubated at 30°C for 1 h. After discarding the liquids in each well, drying the plate, the plate was washed for three times (soaked for 2 min each time) with 200 µl/well wash buffer. After drying the plate, 100 μl HRP–conjugated avidin working solution were added, then the plate was covered with a plate cover, and incubated at 30°C for 1 h. After discarding the liquids and drying the plate, the plate was washed for five times (soaked for 2 min each time) with 200 µl/well wash buffer, then the plate was dried. A total of 90 μl substrate solution was added into each well and the plate was incubated at 37°C for 15–30 min in the dark. Finally, 50 μl of stop solution was added to the wells to terminate the reaction. Five minutes after termination of the reaction, the optical density (OD value) of each well was measured at a wavelength of 450 nm using a microplate reader.

### Statistical analysis

Statistical analyses were performed with SPSS 17.0 software, and the figures were made with GraphPad Prism5 software (GraphPad Software, San Diego, CA). Measurement data are expressed as mean ± SEM. Differences between groups were compared using *t* test and two-way analysis of variance (ANOVA). *P*<0.05 was considered to indicate a significant difference, *P*<0.01 was considered to indicate a highly significant difference, while *P*<0.001 was considered extremely significant.

## Results

### General conditions of rats in NC and DGP groups

**Figure 1:**
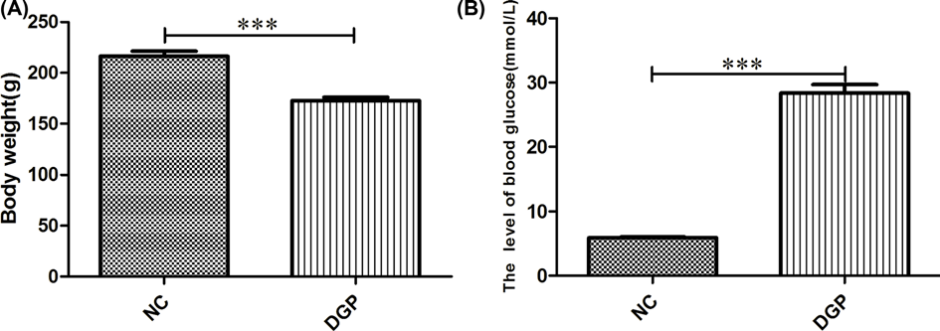
Comparison of body weight and the levels of blood glucose between NC and DGP groups (**A**) Comparison of body weight between the two groups. (**B**) Comparison of the levels of blood glucose between NC and DGP groups. Data were expressed as Mean ± SEM, *n*=10. ***P<0.001, vs control group. NC, normal control group; DGP, diabetic gastroparesis group.

The general conditions of rats in the NC group was good with smooth fur, no significant changes in food and water intake, urine output, amount and shape of stools was observed. Rats in DGP group were abnormally emaciated, and showed loose fur with dull color, hair loss, reduced food intake, loose stools. Compared with NC group, the blood glucose concentrations were significantly higher, while the body weight was significantly lower in the DGP group (Figure 1).

### G**astric smooth muscle cell apoptosis rates in DGP rats**

**Figure 2:**
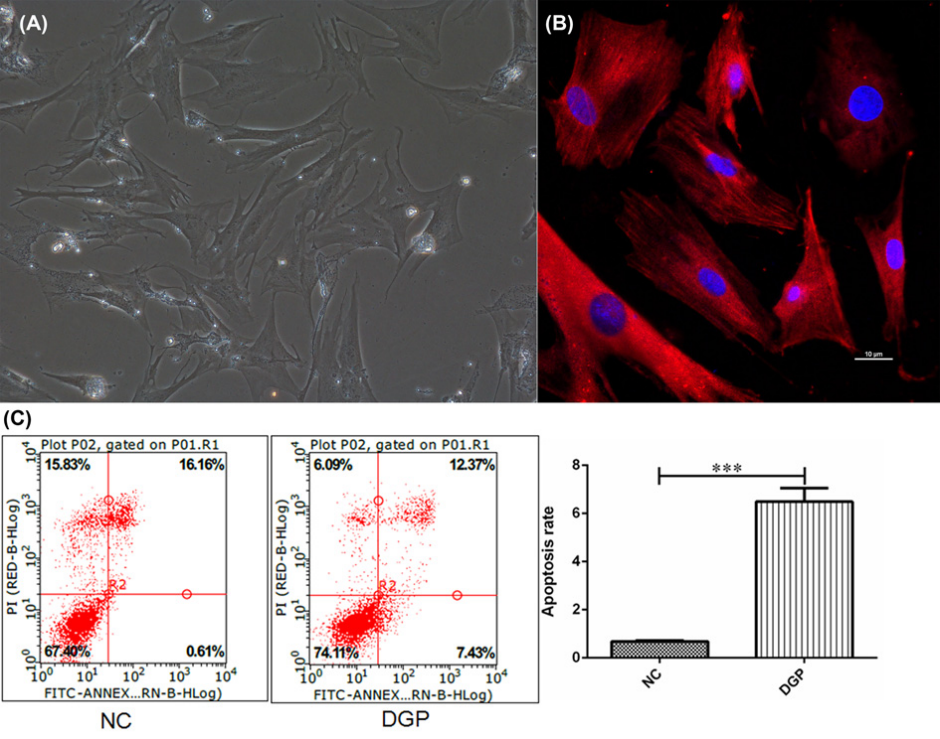
The morphology, α-SMA expression and apoptosis in isolated rat gastric smooth muscle cells (**A**) The morphology of smooth muscle cell. (**B**) The expression of α-SMA in smooth muscle cell (Red fluorescence indicated α-SMA). (**C**) Comparison of cell apoptosis of rat gastric smooth muscle cells between NC and DGP groups. ***P<0.001, vs control group. NC, normal control group; DGP, diabetic gastroparesis group.

Freshly isolated gastric smooth muscle cells were polygonal or fusiform with different lengths under inverted microscope (Figure 2A). Under confocal laser scanning microscopy, the expression of α-SMA was positive in the cytoplasm of smooth muscle cells (Figure 2B). Compared with NC group, the rates of apoptosis in DGP group were significantly increased (0.68 ± 0.03 vs 6.49 ± 0.56, *P*<0.001, respectively) (Figure 2C).

### Phosphorylation changes in AMPK signal pathway in rat gastric smooth muscle tissues of DGP

**Figure 3:**
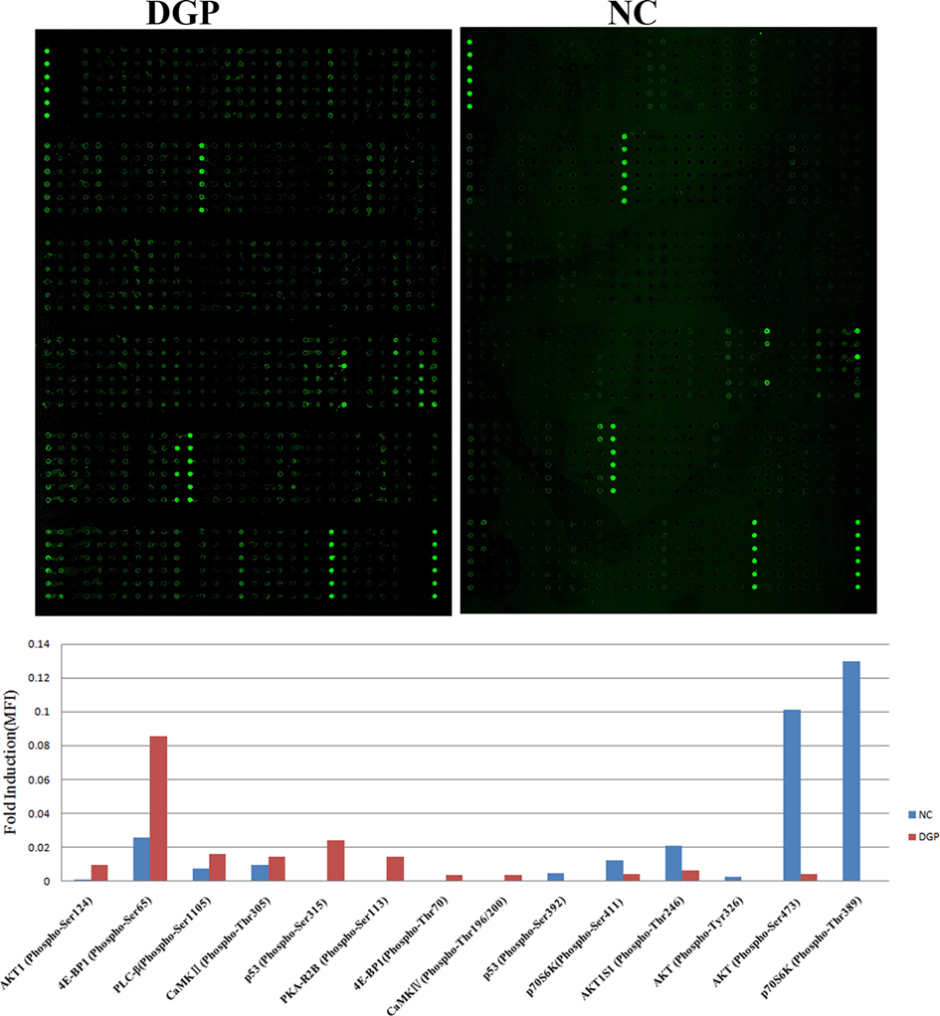
Apoptosis-related differentially phosphorylated proteins in gastric smooth muscle cells between NC and DGP groups NC, normal control group; DGP, diabetic gastroparesis group.

The AMPK Signaling Phospho-Antibody Array that contains 174 highly specific antibodies was used to detect phosphorylation changes in AMPK signal pathway. All target proteins were normalized to internal reference β-actin (i.e. dividing the signal intensity of the target protein with that of β-actin). Based on fold change ≥1.5, positive fold change indicates up-regulation and negative fold change indicates down-regulation. A total of 73 differentially expressed proteins were identified, of which, 30 differentially proteins were identified as phosphorylated. After removing non-specific expression changes, 18 phosphorylated proteins were identified as differentially expressed, among them, 14 differentially phosphorylated proteins were found to be associated with apoptosis, including p53Ser^315^, p53Ser^392^, Akt1Ser^124^, Akt1S1Thr^246^, AktTyr^326^, Akt Ser^473^, p70S6KThr^389^, p70S6KSer^411^, Protein kinase A (PKA)-R2BSer^113^, 4E-BP1Thr^70^, 4E-BP1Ser^65^, PLC-βSer^1105^, CaMKII Thr^305^, CaMKIV Thr^196/200^, which contained 8 proteins, p53, AKT, 4E-BP1, p70S6K, CaMKII, CaMKIV, PLC-β3, PKA (Figure 3).

### Changes of phospho-AMPK/AMPK ratio in rat gastric smooth muscle cells cultured under high-glucose condition

**Figure 4:**
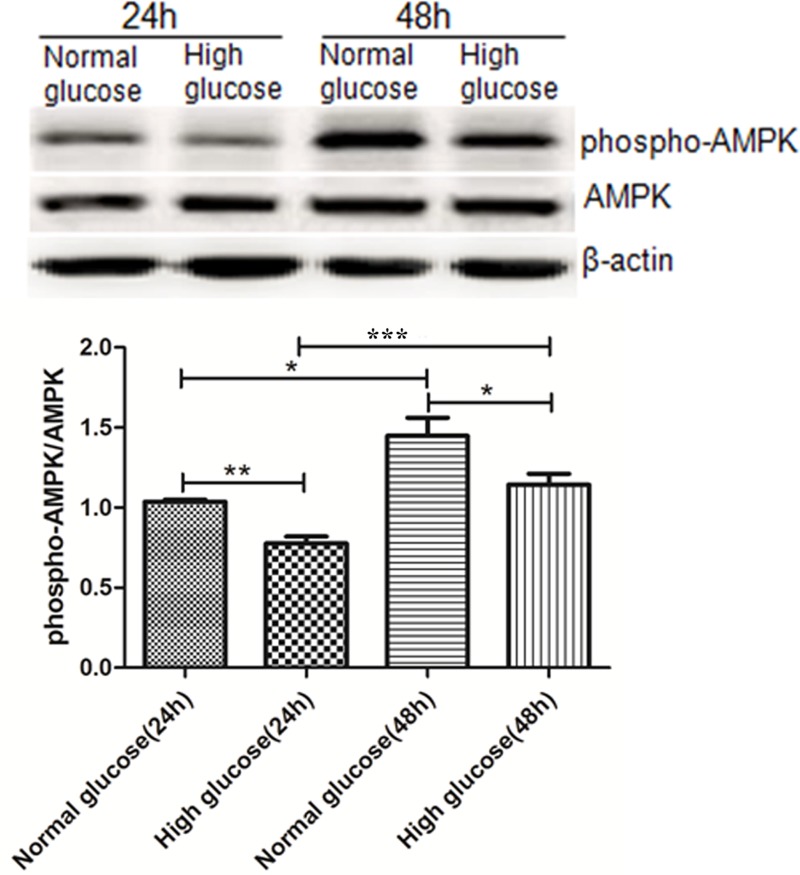
Changes of phospho-AMPK/AMPK in gastric smooth muscle cells of rats in high glucose condition *P<0.05, vs normal glucose-48 h group; ***P<0.05, vs high glucose-48 h group; **P<0.01, vs normal glucose-24 h group.

Phospho-AMPK/AMPK ratio was lower in the high glucose-24h compared with the normal glucose-24 h (1.45 ± 0.11 vs 1.04 ± 0.01, *P*<0.01), as well as in high glucose-48h compared with the normal glucose-48h (1.14 ± 0.07 vs 0.78 ± 0.04, *P*<0.05). Phospho-AMPK/AMPK ratio was higher in the normal glucose-48h compared with the normal glucose-24h (0.78 ± 0.04 vs 1.04 ± 0.01, *P*<0.01), as well as in high glucose-48h compared with the high glucose-48h (1.14 ± 0.07 vs 1.04 ± 0.01, *P*<0.05) (Figure 4).

### Changes in intracellular Ca^2+^ concentrations in rat gastric smooth muscle cells cultured under high glucose condition

**Figure 5:**
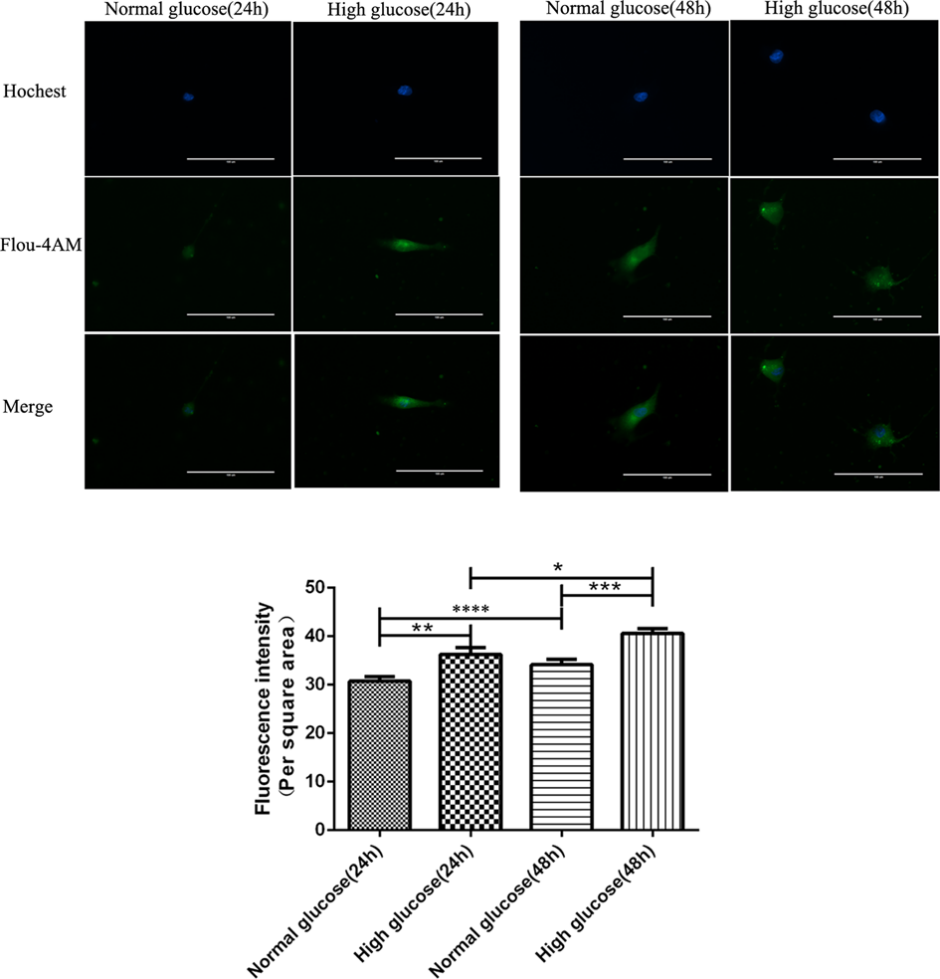
Changes in Ca^2+^ concentrations in rat gastric smooth muscle cells cultured under normal and high glucose conditions *P<0.05, vs normal high glucose-24 h group; **P<0.01, ****P<0.05, vs normal glucose-24 h group; ***P<0.001, vs normal glucose-48 h group.

The changes in intracellular Ca^2+^ concentrations were detected by confocal laser-scanning microscopy, green fluorescence reflected the changes in Ca^2+^ concentrations in rat gastric smooth muscle cells. Five different fields of each group were randomly selected in a double-blinded manner, and the fluorescence intensity was analyzed by Image-Pro Plus 6.0 software. Compared with the high glucose-24h group, significant decrease in the fluorescence intensity was observed in the normal glucose-24h group (36.22 ± 1.43 vs 30.68 ± 0.90, *P*<0.01). Compared with the high glucose-48h group, significant decrease in the fluorescence intensity was observed also observed in the normal glucose-48h group (40.58 ± 0.97 vs 34.09 ± 1.14, *P*<0.001). The fluorescence intensity was significantly decreased in the normal glucose-24h group compared with the normal glucose-48h group (*P*<0.05),which was also decreased in the high glucose-24h group compared with the high glucose-48h group (*P*<0.05) (Figure 5).

### Transfection efficiency of RNA interference-mediated silencing of AMPK

**Figure 6:**
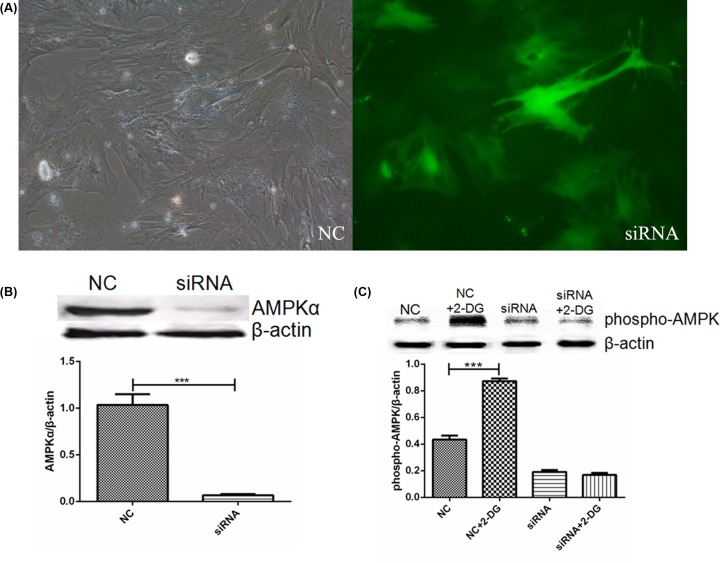
siRNA transfection efficiency in rat gastric smooth muscle cells (**A**) Transfection efficiency of siRNA after 72 h of transfection (×200). (**B**) AMPKα expression in NC and siRNA groups after silencing of AMPK. ***P<0.001, vs control group**.** (**C**) Changes in AMPK phosphorylation after silencing of AMPKα and adding 2-DG. ***P<0.001, vs control group. NC, normal control group; siRNA, siRNA group.

The transfection efficiency of siRNA for cells was more than 70% at 72 h after transfection. After the cells were transfected with AMPKα1/α2 siRNA, the AMPKα expression was higher in NC group compared with the siRNA group (1.03 ± 0.12 vs 0.06 ± 0.01; *P*<0.001), indicating that AMPKα expression was significantly inhibited. After adding 2-DG, the expression of phospho-AMPK was higher in NC+2-DG groups compared with the NC groups (0.43 ± 0.03 vs 0.87 ± 0.02; *P*<0.001), the results indicated that phospho-AMPK expression was not significantly changed after transfection with AMPKα1/α2 siRNA (Figure 6).

### Expression of p53

**Figure 7:**
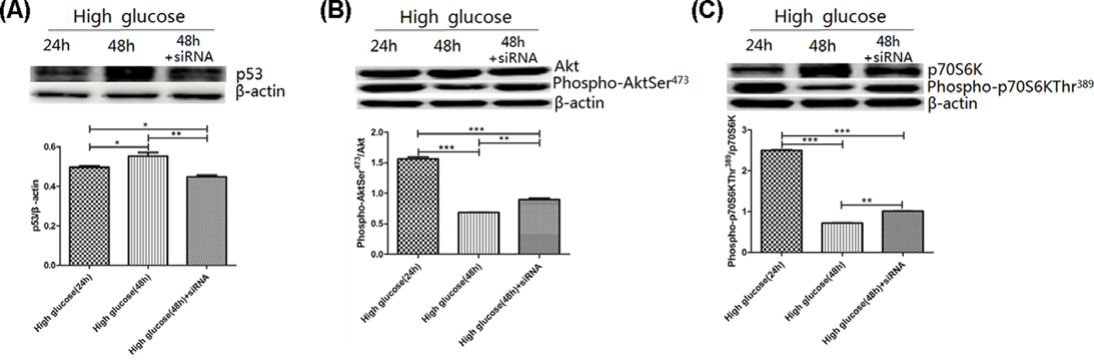
p53 expression, Akt and p70S6K activities in rat gastric smooth muscle cells under high glucose and AMPK silencing conditions (**A**) Expression of p53 in rat gastric smooth muscle cells under high glucose and AMPK silencing conditions. *P<0.05, vs high glucose-24h group; **P<0.01, vs high glucose-48h group. (**B**) Expression of Akt and phospho-AktSer^473^ in rat gastric smooth muscle cells under high glucose and AMPK silencing conditions. ***P<0.001, vs the high glucose-24h group, **P<0.001, vs the high glucose-48h group. (**C**) Expression of p70S6K and phospho-p70S6KThr^389^ in rat gastric smooth muscle cells under high glucose and AMPK silencing conditions. ***P<0.001, vs high glucose-24h group, **P<0.001, vs high glucose-48h group.

p53 expression was higher in the high glucose-48h group (0.55 ± 0.02) compared with the high glucose-24h group (0.5 ± 0.02) and high glucose-48h+siRNA group (0.45 ± 0.01) (*P*<0.05) (Figure 7A).

### Activity of Akt

Akt activity was expressed as the ratio of phospho-AktSer^473^ to total Akt. The ratio of phospho-AktSer^473^/Akt was higher in the high glucose-24h group (1.56 ± 0.03) compared with high glucose-48h group (0.69 ± 0.01) and high glucose-48h+siRNA group (0.9 ± 0.02) (*P*<0.001). The high glucose-48 h group had lower ratio of phospho-AktSer^473^/Akt compared with high glucose-48h+siRNA group (*P*<0.001) (Figure 7B).

### Activity of p70S6K

p70S6K activity was expressed as the ratio of phospho-p70S6KThr^389^ to total p70S6K. The ratio of phospho-p70S6KThr^389^/p70S6K was lower in the high glucose-48h group (0.72 ± 0.01) and high glucose-48h+siRNA group (1.02 ± 0.01) compared with the high glucose-24h group (2.5 ± 0.02, *P*<0.001), which was also lower in the high glucose-48h group compared with the high glucose-48h+siRNA group (*P*<0.001) (Figure 7C).

### Expression of Bcl-2, BAX and Caspase-3

**Figure 8:**
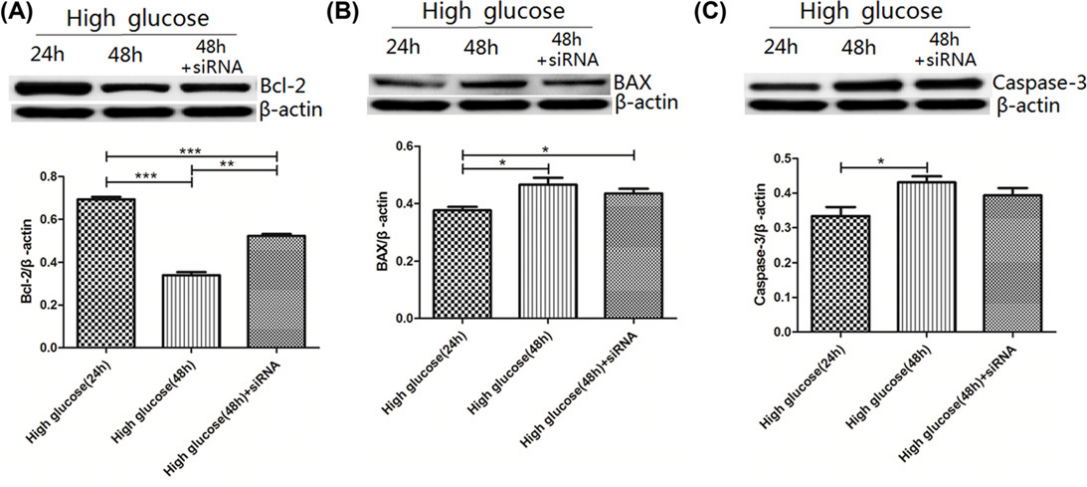
Expression of Bcl-2, BAX and Caspase-3 in rat gastric smooth muscle cells under high glucose and AMPK silencing conditions (**A**) Expression of Bcl-2 in rat gastric smooth muscle cells under high glucose and AMPK silencing conditions. ***P<0.001, vs high glucose-24h group, **P<0.001, vs the high glucose-48h group. (**B**) Expression of BAX in rat gastric smooth muscle cells under high glucose and AMPK silencing conditions. *P<0.05, vs high glucose-24h group. (**C**) Expression of Caspase-3 in rat gastric smooth muscle cells under high glucose and AMPK silencing conditions. *P<0.05, vs high glucose-24h group.

Compared with the high glucose-24h group (0.69 ± 0.01), Bcl-2 expression was lower in the high glucose-48h group (0.34 ± 0.01) and high glucose-48h+siRNA group (0.52 ± 0.01) group (*P*<0.001, respectively). The high glucose-48h+siRNA group showed decreased expression of Bcl-2 compared with the high glucose-48h group (*P*<0.001). Compared with the high glucose-24h group (0.38 ± 0.01), BAX expression was higher in the high glucose-48h (0.47 ± 0.02) and high glucose-48h+siRNA (0.44 ± 0.02) group (*P*<0.001, *P*<0.05, respectively). T had lower BAX expression which was lower in the high glucose-48h+siRNA group compared with the high glucose-48h group, but there was no significant difference between the two groups. Caspase-3 expression was higher in the high glucose-48h group (0.43 ± 0.02) compared with high glucose-24h group (0.33 ± 0.03) (*P*<0.05). Caspase-3 expression were increased in the high glucose-48h, and decreased in the high glucose-24h group compared with the high glucose-48h +siRNA group (0.39 ± 0.02), however, no significant difference was observed between the high glucose-48h+siRNA group and high glucose-48h, high glucose-24h group (Figure 8).

### Expression of 4E-BP1 and PLC-β3

**Figure 9:**
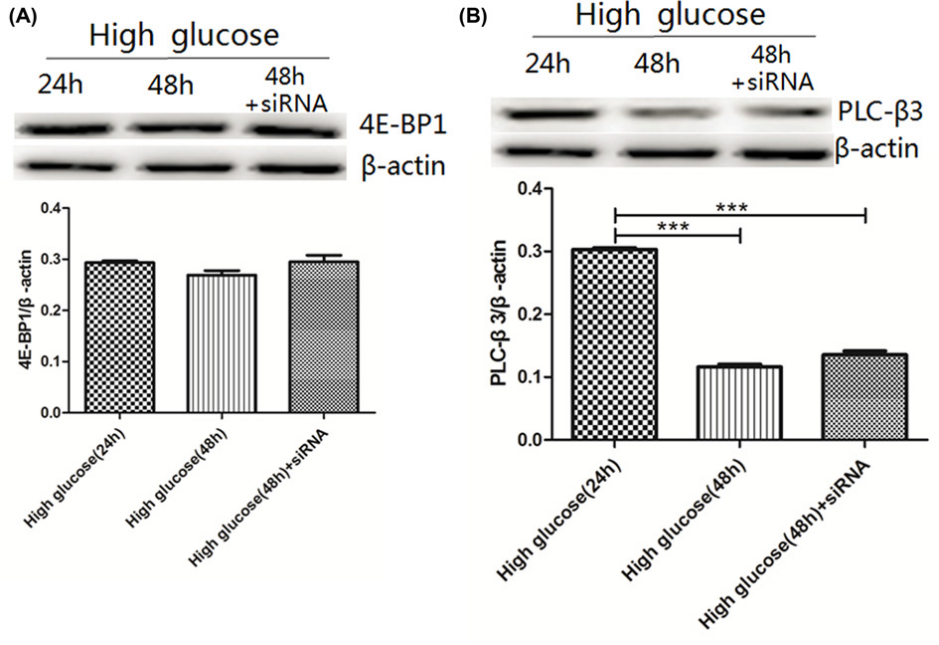
Expression levels of 4E-BP1 and PLC-β 3 in rat gastric smooth muscle cells under high glucose and AMPK silencing conditions (**A**) Expression of 4E-BP1 in rat gastric smooth muscle cells under high glucose and AMPK silencing conditions. (**B**) Expression of PLC-β3 in rat gastric smooth muscle cells under high glucose and AMPK silencing conditions. ***P<0.001, vs the high glucose-24h group.

4E-BP1 expression in the high glucose-24h, high glucose-48h and high glucose-48h+siRNA groups were 0.29 ± 0.01, 0.27 ± 0.01, 0.3 ± 0.01, respectively, there was no significant difference among of PLC-β3 was lower in the high glucose-48h (0.12 ± 0.01) and high glucose-48h+siRNA (0.14 ± 0.01) groups (*P*<0.001). There was no significant difference between the high glucose-48h and high glucose-48h+siRNA groups (Figure 9).

### Activity of CaMKII

**Figure 10:**
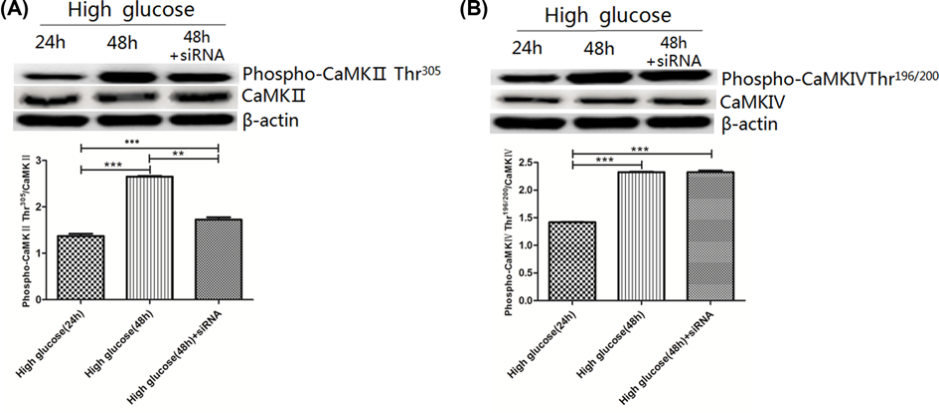
CaMKII and CaMKIV activities in rat gastric smooth muscle cells under high glucose and AMPK silencing conditions (**A**) Expression of CaMKII and phospho-CaMKIIThr^305^ in rat gastric smooth muscle cells under high glucose and AMPK silencing conditions. ***P<0.01, vs the high glucose-24h group; **P<0.001, vs high glucose-48h group. (**B**) Expression of CaMKIV and phospho-CaMKIVThr^196/200^ in rat gastric smooth muscle cells under high glucose and AMPK silencing conditions. ***P<0.001, vs the high glucose-24h group.

CaMKII activity was expressed as the ratio of Phospho-CaMKII Thr^305^ to total CaMKII. The ratio of Phospho-CaMKII Thr^305^/CaMKII was higher in the high glucose-48h (2.65 ± 0.02) and high glucose-48h+siRNA (1.72 ± 0.05) groups compared with the high glucose-24h group (1.37 ± 0.05, *P*<0.01),which was also higher in the high glucose-48h group compared with the high glucose-48h+siRNA group (*P*<0.01) (Figure 10A).

### Activity of CaMKIV

CaMKIV activity was expressed as the ratio of Phospho-CaMKIV Thr^196/200^ to total CaMKIV. The ratio of Phospho-CaMKIVThr^196/200^/CaMKIV was higher in the high glucose-48h (2.33 ± 0.01) and high glucose-48h+siRNA (2.33 ± 0.03) groups compared with the high glucose-24h group (1.42 ± 0.01, *P*<0.001), there was no significant difference between high glucose-48h and high glucose-48h+siRNA groups (Figure 10B).

### PKA activity detected by enzyme-linked immunosorbent assay method

**Figure 11:**
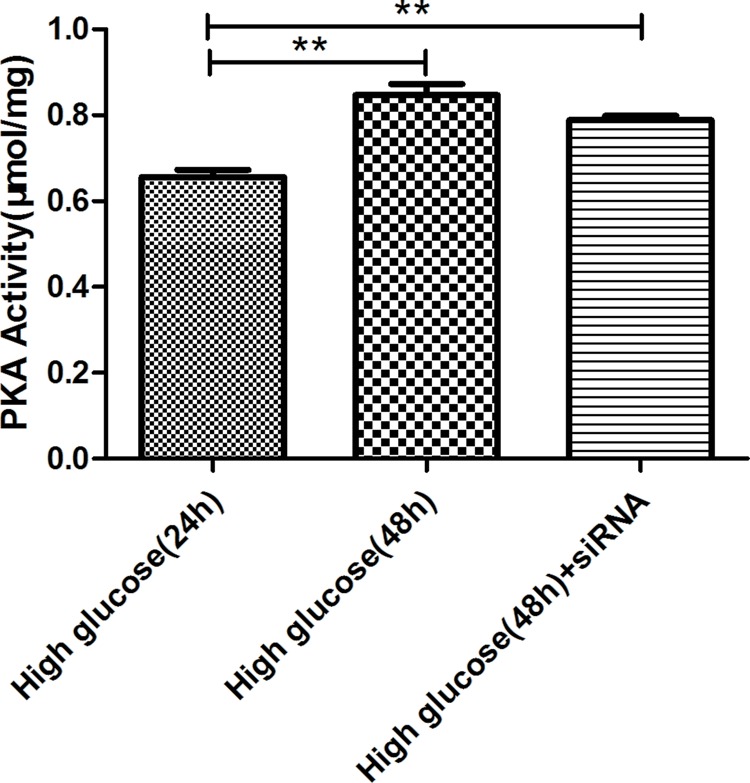
Activity of the PKA in rat gastric smooth muscle cells under high glucose and AMPK silencing conditions **P<0.01, vs high glucose-24h group.

PKA activity was higher in the high glucose-48h (0.85 ± 0.03) and high glucose-48h+siRNA (0.79 ± 0.01) groups compared with the high glucose-24h group (0.66 ± 0.02, *P*<0.001). There was no significant difference between high glucose-48h and high glucose-48h+siRNA groups (Figure 11).

## Discussion

DGP is mainly manifested as delayed gastric emptying due to the decreased contraction of gastric smooth muscle. It is considered that high glucose-induced changes in number of gastric smooth muscle cells may be the major cause of DGP. In order to explore the effect of AMPK on apoptosis of gastric smooth muscle cells in DGP rats and the underlying mechanism, we first prepared a rat model of DGP diabetes, and observed the apoptosis of gastric smooth muscle cells in DGP rats, the results showed that the apoptosis rate of gastric smooth muscle cells in DGP rats was significantly higher than that in the normal control group, which suggested that apoptosis is involved in the development of DGP. Our previous study [1] showed that changes in AMPK activity is involved in the occurrence of DGP, and AMPK can regulate, but the specific regulatory mechanisms underlying DGP devlopment are still unclear. Therefore, we used AMPK Signaling Phospho-Antibody Array to observe the phosphorylation changes in AMPK signal pathway, a total of 14 apoptosis-related differentially phosphorylated proteins were found, which contained eight proteins, p53, AKT, 4E-BP1, p70S6K, CaMKII, CaMKIV, PLC-β3 and PKA-R2B.

Then we cultured rat gastric smooth muscle cells *in vitro*, and observed changes in AMPK activity and intracellular Ca^2+^ concentrations in rat gastric smooth muscle cells. The results showed that AMPK activity was higher in normal glucose-48h group than that in normal glucose-24h group, which was also higher in high glucose-48h group than that in high glucose-24h group, but AMPK activity was lower in high glucose-24h group than that in normal glucose-24h group, as well as in high glucose-48h group than that in normal glucose-48h group, the results indicated that AMPK activity was gradually increased with time prolonged under either normal nor high glucose conditions, the AMPK activity was increased in a time-dependent manner. It is interesting that AMPK activity was lower under the high glucose condition than under the normal glucose condition, this findings suggest that high glucose may activate certain mechanisms exist in the cell which has delaying or inhibitory effects on the activation of AMPK, However, AMPK is still activated with the activation of upstream AMPK kinases. The inhibitory effect is gradually weakened with time, and the mechanisms involved need to be further investigated.

We detected the changes in concentration of Ca^2+^ in *in vitro*-cultured rat gastric smooth muscle cells under high-glucose conditions. The results showed that significant changes in the concentration of Ca^2+^ was observed in each group, Ca^2+^ concentration was increased in high glucose group than that in normal glucose group after both 24 and 48 h of culture. Ca^2+^ concentration was also increased in rat gastric smooth muscle cells cultured under high glucose and normal glucose conditions for 48 h compared with those cultured for 24 h. This result indicates that high glucose can promote the increase in Ca^2+^ concentration in rat gastric smooth muscle cells. After silencing AMPK in rat gastric smooth muscle cells by RNA interference, we detected the transfection efficiency and silencing efficiency, and divided the cells into high glucose+24h group, high glucose+48h group and high glucose+48h + siRNA group, and then verified the apoptosis**-**related protein expression levels.

P53 is a tumor suppressor protein, which can regulate the expression of several genes that maintain homeostasis, including cell-cycle regulation, redox homeostasis, DNA replication and repair, apoptosis and autophagy, energy metabolism [4,5]. P53 is closely associated with AMPK. P53 transcription activates Sestrin1/2, which then activates AMPK. AMPK activation, in turn, increases p53 expression through transcription and induces p53 stabilization via direct phosphorylation of p53. Okoshi et al. [6] found that AMPK activation phosphorylates p53Ser^46^ and leads to accumulation of p53. Nieminen et al. [7] found that AMPK activation can cause p53Ser^15^ phosphorylation and p53 accumulation. Murine double-minute 2 (MDM2) is a major inhibitor of p53 and mediates p53 degradation. Phosphorylation of p53 prevents the inhibitory effect of Mdm2, thereby inhibiting the degradation of p53 protein. This implies that p53 phosphorylation indirectly promots p53 expression. Results from AMPK Signaling Phospho-Antibody Array showed that high glucose-induced AMPK activation caused the differential expression of two phosphorylation sites of p53 in rat gastric smooth muscle cells, of which, p53Ser^315^ expression were up-regulated, and p53Ser^392^ expression was down-regulated. To verify whether high glucose-induced AMPK activation can affect p53 expression through these two phosphorylation sites, we used Western blot assay to detect p53 expression in high glucose-24, high glucose-48h and high glucose 48h+siRNA groups, the results showed that expression of p53 was increased in wild-type cells of the high glucose-48h group, but was decreased after silencing AMPK. This result indicates that high glucose-induced AMPK activation can induce accumulation of p53 protein and promote apoptosis through up-regulating p53Ser^315^ expression and down-regulating p53Ser^392^ expression. A study found that p53 can up-regulate the expression of pro-apoptotic BAX and inhibit the transcription of Bcl-2, and can also promote the release of cytoC from mitochondria to activate Caspase3 and promote apoptosis [8].

Akt is a serine/threonine-specific protein kinase, consists of 480 amino acid residue. Akt activity is regulated by its phosphorylation (Phospho-AktSer^473^/Akt ratio), whereas activated Akt regulates cell cycle transition, expression of proapoptotic and anti-apoptotic genes by phosphorylating downstream substrates [9]. Studies showed that activated Akt can phosphorylate Bad at Ser^136^ and lead to the dissociation of BAD from Bcl-2, Bcl-xL, and restore the anti-apoptotic effect of Bcl-2 and Bcl-xL, and also stabilize the mitochondrial membrane potential and block the release of cytochrome *c*. Activated Akt can cause caspase3 inactivation and terminate the antagonistic effect of forkhead on Bcl-2 or Bcl-xL in outer mitochondrial membrane, the anti-apoptotic function of Bcl-2 and Bcl-xL was restored after releasing, which can also inhibit p53 accumulation and thus inhibit apoptosis [10]. AMPK has regulatory effect on Akt phosphorylation, but research findings remain inconsistent across studies. King et al. [11] found that activated AMPK reduces Akt phosphorylation in hippocampal neurons. The same conclusion was reached by Rattan et al., they found that activated AMPK reduces Akt phosphorylation in tumor cells [12]. However, in a study investigating the effects of alcoholic intake on myocardium, Lang and Korzick [13] found that activated AMPK does not alter Akt phosphorylation. The above results indicated that the regulatory effect of AMPK activation on Akt phosphorylation is different under the action of different influencing factors. In the present study, results from AMPK Signaling Phospho-Antibody Array showed that under high-glucose condition, activated AMPK caused the differential expression of four phosphorylation sites of Akt in rat gastric smooth muscle cells, Akt1Ser^124^ expression was up-regulated, whereas Akt1S1Thr^246^, AktTyr^326^, AktSer^473^ expression was down-regulated. To verify the effect of these four phosphorylation sites on Akt activity, we used Western blot assay to detect the changes in phospho-AktSer^473^ and total Akt, and calculated the Phospho-AktSer^473^/Akt ratio in high glucose-24 group, high glucose-48h group and high glucose-48h+siRNA group, we found that Akt activity was decreased in the high glucose-48h group, which was increased after silencing AMPK, indicating that under high-glucose condition, activated AMPK can inhibit Akt activity by regulating the expression of Akt1Ser^124^, Akt1S1Thr^246^, AktTyr^326^, Akt Ser^473^, and then promote rat gastric smooth muscle cell apoptosis by regulating downstream substrates (Bad and Bcl-2) of Akt.

p70S6K and 4E-BP1 are the downstream substrates of AMPK [14,15]. Study found that AMPK can down-regulate p70S6K activity and inhibit small GTPase Rheb (Ras-homolog enriched in brain) through the TSC-1/TSC-2 complex [16]. p70S6K activity is regulated by the phosphorylation of p70S6K, activated p70S6K can phosphorylate Bad at Ser^136^ and lead to the dissociation of BAD from Bcl-2, Bcl-xL [17]. In the present study, results from AMPK Signaling Phospho-Antibody Array showed that under high-glucose condition, activated AMPK caused the differential expression of two phosphorylation sites (Ser^41^ and Thr^389^) of p70S6K in rat gastric smooth muscle cells, expression of p70S6KSer^411^ and p70S6KThr^389^ were both decreased. Phosphorylation at Thr^389^ is critical for p70S6K activation, so the degree of phosphorylation at this site can reflect the activity of p70S6K (Phospho- p70S6KThr^389^/p70S6K). Phosphorylation at Ser^411^ is beneficial to the release of self-suppression of C-terminal of p70S6K, facilitating the activation of p70S6K. To verify whether activated AMPK under high-glucose condition can affect the p70S6K activity through these two phosphorylation sites, we used Western blot assay to detect the expression of phospho-p70S6K Thr^389^ and p70S6K, and calculated the Phospho-p70S6KThr^389^/p70S6K ratio in high glucose-24 group, high glucose-48h group and high glucose-48h+siRNA group, and found that p70S6K activity was increased in the high glucose-48h group, and was decreased after silencing AMPK. The results suggested that under high glucose condition, activated AMPK can inhibit the phosphorylation of p70S6K^Thr389^ and p70S6K^Ser411^, and down-regulate p70S6K activity, which further regulate Bcl-2 and Bcl-xL to promote rat gastric smooth muscle cell apoptosis.

PKA belongs to the Ser/Thr protein kinase family, which is highly dependent on cAMP. Under pathological conditions, increased cAMP can cause excessive up-regulation of PKA, which can block Akt and p70S6K phosphorylation, down-regulate Mcl-1, c-Myc and Hedgohog signaling pathways, up-regulate BIM, kinome, JNK and NF-κB, thereby regulating BAX, Bcl-2 and Caspase3 expression, and promoting apoptosis [18]. In the present study, results from AMPK Signaling Phospho-Antibody Array showed that under high-glucose condition, activated AMPK caused the differential expression of one phosphorylation site of PKA in rat gastric smooth muscle cells, expression of PKA-R2BSer^113^ was up-regulated. To verify whether PKA activity was affect by AMPK activation through this phosphorylation site under high-glucose condition, we used enzyme-linked immunosorbent assay (ELISA) to detect the PKA activity in high glucose-24 group, high glucose-48h group and high glucose-48h+siRNA group, the results showed that PKA activity was increased in the high glucose-48h group, but changes in PKA activity was not obvious after silencing AMPK. Those results suggested that under high glucose condition, AMPK can increase the PKA activity by phosphorylating PKA-R2BSer^113^, but the effect of AMPK on PKA activity is not decisive. Interestingly, increased PKA activity is also beneficial to AMPK activation [19]. In addition, PKA can increase intracellular Ca^2+^ concentration, and promote cell contraction through phosphorylating Ca^2+^ channels. Combining with our results that intracellular free Ca^2+^ was increased, we considered that increase PKA activity is also involved in the increase in intracellular free Ca^2+^.

P53, Akt, p70S6K or PKA can both affect cell apoptosis through regulating BAX, Bcl-2 and Caspase-3. So, in the present study, we detected the expression of BAX, Bcl-2 and Caspase3 in the high glucose-24h, high glucose-48h and high glucose-48h+siRNA groups, and found that BAX and Caspase-3 expression were increased in the high glucose-48h group, which were decreased after silencing AMPK, but the decrease was not obvious. Bcl-2 expression was decreased in the high glucose-48h group, which was increased after silencing AMPK, indicating that high glucose-induced AMPK activation can regulate BAX, Bcl-2 and Caspase-3 expression through p53, Akt, p70S6K, PKA pathways, and its regulatory effect on Bcl-2 expression is more obvious.

4E-BP1 can bind to eIF4E, thereby inhibiting the translational effects of eIF4E on anti-apoptotic proteins. 4E-BP1 has seven phosphorylation sites, including Thr^37^, Thr^46^, Ser^65^, Thr^70^, Ser^83^, Ser^101^ and Ser^112^, the former four phosphorylation sites are associated with AMPK-mTOR pathway. Upon stimulation by upstream stimulating factors, 4E-BP1 is first phosphorylated at Thr^37^ and Thr^46^, this is the necessary conditions for the phosphorylation of 4E-BP1Thr^70^ and 4E-BP1Ser^65^ [20]. However, some scholars considered that phosphorylation at Thr^37^ and Thr^46^ sites alone can not promote the dissociation of 4E-BP1 from eIF4E, and also phosphorylation at Ser^65^ and Thr^70^ sites alone can not block the binding of 4E-BP1 to eIF4E. Therefore, phosphorylation at both the four sites Thr^37^, Thr^46^, Ser^65^, Thr^70^ promotes the dissociation of of eIF4E/4E-BP1 [21]. In the present study, results from AMPK Signaling Phospho-Antibody Array showed that under high-glucose condition, activated AMPK caused the differential expression of two phosphorylation sites (Ser^65^ and Thr^70^) in 4E-BP1 in rat gastric smooth muscle cells, expression of 4E-BP1Ser^65^ and 4E-BP1Thr^70^ were both up-regulated. To verify whether 4E-BP1 expression was affect by AMPK activation through these two phosphorylation sites under high-glucose condition, we used Western blot assay to detect the expression of 4E-BP1 in high glucose-24, high glucose-48h and high glucose-48h+siRNA groups, and no significant change in 4E-BP1 expression was found in each group. Those results indicated that under high-glucose condition, AMPK activation can not cause the dissociation of eIF4E/4E-BP1, thereby affecting the translation of anti-apoptotic proteins and indirectly promoting apoptosis. Meanwhile, the results also showed that phosphorylation at both Ser^65^ and Thr^70^ sites under high-glucose condition could not block the binding of 4E-BP1 to eIF4E, which are consistent with literature findings.

PLC is widely distributed in tissues, the PLC subtypes PLC-β3 is mainly present in the gastrointestinal tract. PLCβ/inositol trisphosphate (IP3)/Ca^2+^ is a common pathway associated with PLC. After IP3 was generated, IP3 diffuses from the plasma membrane into the cytoplasm and binds to the IP3R-Ca^2+^ channel on the endoplasmic reticulum/sarcoplasmic reticulum. The IP3R-Ca^2+^ channel is open after binding to IP3, which promotes the rapid increase in Ca^2+^ levels in the cytoplasm. The binding of IP3 to IP3R-Ca^2+^ channel is selective and saturable, and is affected by cytoplasmic Ca^2+^ and pH. An increased cytoplasmic Ca^2+^ level can reduce the affinity of IP3 and IP3R. The increased cytoplasmic pH level can increase the affinity of IP3 and receptor IP3R, and enhance the Ca^2+^ mobilization from the endoplasmic/sarcoplasmic reticulum. IP3 can then be phosphorylated to IP4 by Ca^2+^/CaM-dependent IP3 kinase. Once IP3 completes its signaling function, high Ca^2+^ levels can be pumped out of the cytoplasm to the cell exterior through the Ca^2+^-pumping ATPase (Ca^2+^-ATPase) on the plasma membrane, or intracytoplasmic Ca^2+^ concentration was reduced through the endoplasmic/sarcoplasmic reticulum Ca^2+^ pump. Increase in phospho-PLC-βSer^1105^ expression can inhibit PLC-β3 expression [22]. In the present study, results from AMPK Signaling Phospho-Antibody Array showed that under high glucose condition, activated AMPK caused the differential expression of one phosphorylation sites (Ser^1105^) in PLC-β in rat gastric smooth muscle cells, expression of PLC-βSer^1105^ were up-regulated. To verify whether PLC-β3 expresson was affected by AMPK activation through the phosphorylation site under high-glucose condition, we used Western blot assay to detect the expression of PLC-β3 in high glucose-24 group, high glucose-48h group and high glucose-48h+siRNA group, we found that PLC-β3 expression was decreased in the high glucose-48h group, but the change in PLCβ3 expression was not obvious after silencing AMPK. This result indicated that under high-glucose condition, activated AMPK can down-regulate PLC-β3 expression by increasing phosphorylation of PLC-βSer^1105^, but it regulatory effect is not obvious. Decreased PLC-β3 expression can reduce IP3 generation, reduce Ca^2+^ influx and inhibit smooth muscle contraction. Interestingly, increased free Ca^2+^ levels in rat gastric smooth muscle cells under high glucose was found in our aforementioned results. This finding suggested that decreased PLC-β3 expression may also be involved in the increase in free Ca^2+^ levels in rat gastric smooth muscle cells. This may be because that PLC-β3 expression was gradually decreased under high-glucose condition in a time-dependent manner, which leads to the imbalance of Ca^2+^ influx and outflow (influx > outflow) and the imbalance of endoplasmic/sarcoplasmic reticulum restoration and release (release > restoration), so from the macroscopic view, the smooth muscle contraction was inhibited, and from the microscopic view, intracellular free Ca^2+^ was increased in gastric smooth muscle cells.

Ca^2+^/CaM-dependent protein kinase (CaMK) is a Ser/Thr protein kinase, which has two types, one is a specific functional type, such as CaMKIII, myosin light chain kinase (MICK), and elongation factor-2 kinase (e2F), which can up-regulate muscle contraction, glycogenolysis and protein synthesis; another is multifunctional type, including CaMKI, II, IV and V. CaMKII activation is achieved by releasing the self-inhibition of the pseudosubstrate domain. At rest condition, the self-inhibitory domain (pseudosubstrate domain) masks the catalytic domain, prevents the substrate from binding to ATP and prevents substrate phosphorylation, which allowing CaMKII to stay in its own inhibitory state. The increase in cytoplasmic Ca^2+^ concentration promotes the binding of Ca^2+^/CaM to the CaM-binding domain of CaMKII, resulting in conformational changes of CaMKII, and unmasking of the catalytic domain from the pseudosubstrate domain, thereby activating autophosphorylation of CAMKII. CaMKIIThr^305^, CaMKIIThr^286^ and CaMKIIThr^306^ are autophosphorylation sites of the CaMKII, increased phosphorylation levels of CaMKII can promote CaMKII activation (CaMKIIThr^305^/CaMKII) [23]. CaMKII activation can phosphorylate Ca^2+^ channels (ryanodine receptor/IP3R) on the sarcoplasmic/endoplasmic reticulum, and promote pumping out of Ca^2+^. CaMKII activation can also cause the phosphorylation of phosphoproteins, and activation of sarcoplasmic reticulum Ca^2+^-ATPase, which increase Ca^2+^ uptake recovery. Study found that CaMKII is a key protein regulating the concentration of intracellular Ca^2+^. Increased CaMKII activity can lead to intracellular Ca^2+^ accumulation and cause apoptosis [24]. In the present study, results from AMPK Signaling Phospho-Antibody Array showed that under high-glucose condition, AMPK activation caused the differential expression of one phosphorylation site in CaMKII in rat gastric smooth muscle cells, expression of CaMKII Thr^305^ was up-regulated. To verify whether CaMKII activity was affect by AMPK activation through this phosphorylation site under high glucose condition, we used Western blot analysis to detect Phospho-CaMKII Thr^305^/CaMKII ratio in high glucose-24 group, high glucose-48h group and high glucose-48h+siRNA group, we found that Phospho-CaMKII Thr^305^/CaMKII ratio was increased in high glucose-48h group and was decreased after silencing AMPK. This result indicates that under high-glucose condition, AMPK activation can increase CaMKII activity by phosphorylating the CaMKIIThr^305^ site, thereby participating in the increase in intracellular free Ca^2+^.

The function of CaMKIV is similar to CaMKII, but its structure is different from that of CaMKII. CaMKIV has an ‘activation loop’. The binding of CaMKIV to Ca^2+^/CaM can expose this ‘activation loop’, which is then phosphorylated by upstream CaMKK [25]. Study suggests that CaMKK phosphorylation is required for CaMKIV activation [26]. CaMKIVThr^196/200^ is a site in the loop, the degree of CaMKIVThr^196/200^ phosphorylation reflects CaMKIV activation level. CaMKIV is closely related to apoptosis [27]. In the present study, results from AMPK Signaling Phospho-Antibody Array showed that under high-glucose condition, activated AMPK caused the differential expression of one phosphorylation site in CaMKIV in rat gastric smooth muscle cells, expression of CaMKIV Thr^196/200^ was up-regulated. To verify whether CaMKIV activity was affect by AMPK activation through this phosphorylation site under high-glucose condition, we used Western blot assay to detect the Phospho-CaMKIV Thr^196/200^/CaMKIV ratio in high glucose-24 group, high glucose-48h group and high glucose-48h+siRNA group, the results showed that Phospho-CaMKIVThr^196/200^/CaMKIV ratio was increased in high glucose-48h group, but no obvious change in Phospho-CaMKIV Thr^196/200^/CaMKIV ratio was found after silencing AMPK. This result indicates that under high-glucose condition, AMPK activation can increase CaMKIV activity by phosphorylating the CaMKIVThr^196/200^ site, but its effects is not obvious. Up-regulation of CaMKIV activity is involved in the increase in intracellular free Ca^2+^.

PKA, PLC-β3, CaMKII and CaMKIV can both increase intracellular Ca^2+^ concentrations, and the results of our study confirmed that Ca^2+^ concentrations were increased in rat gastric smooth muscle cells cultured under high-glucose conditions. It is well known that Ca^2+^ can induce endoplasmic reticulum stress and initiate endoplasmic reticulum stress apoptotic signaling pathway, which can also stimulate mitochondria, and initiate mitochondria apoptotic signaling pathway. This result indicates that under high-glucose condition, activated AMPK can induce the increase in Ca^2+^ concentrations in rat gastric smooth muscle cells, and promote cell apoptosis through regulating the expression of PKA, PLC-β3, CaMKII and CaMKIV.

**Figure 12:**
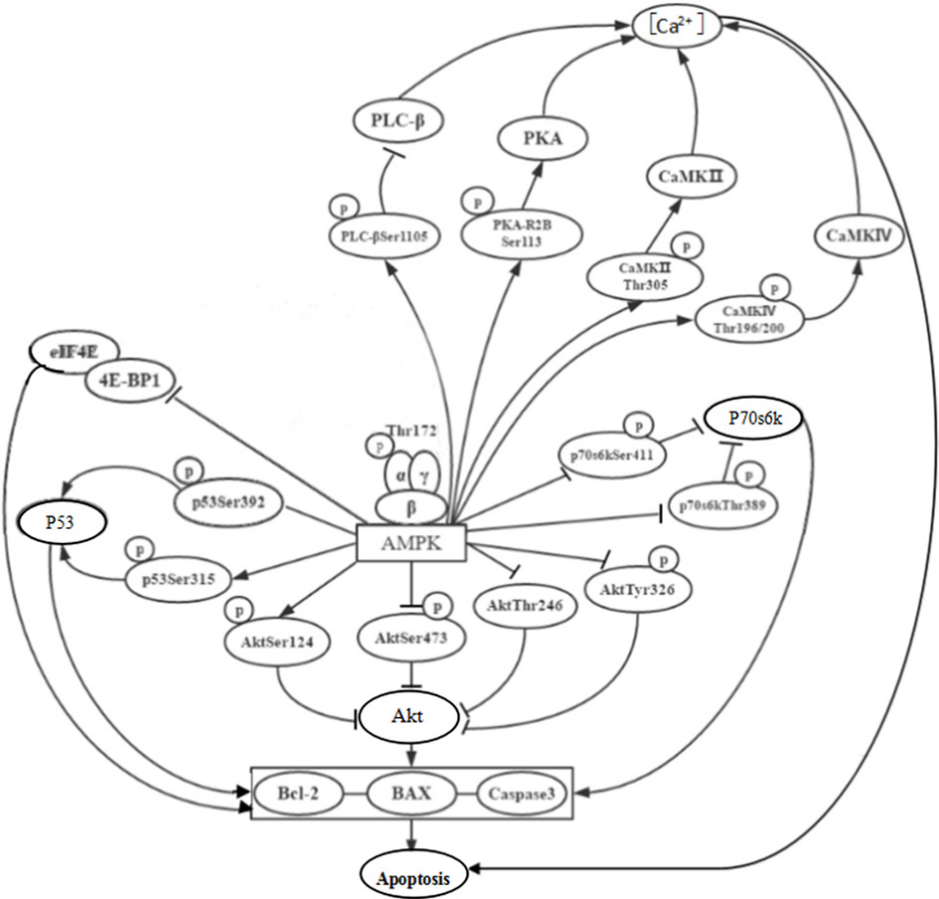
Molecular mechanism of AMPK-mediated apoptosis of rat gastric smooth muscle cells cultured under high-glucose condition

Taken together, our finding suggested that under high-glucose condition, activated AMPK directly or indirectly promotes apoptosis by regulating the expression and activity of p53, Akt, p70S6K, PKA, PLC-β3, CaMKII, CaMKIV and 4E-BP1 in rat gastric smooth muscle cells (Figure 12). Further studies are needed to confirm the results by investigating the effect of AMPK on bax expression and bcl-2/BAX ratio in gastric smooth muscle cells of DGP rats, and inhibiting AMPK expression with AMPK inhibitors.

## Abbreviations

AMPK: AMP-activated protein kinase
BAX: Bcl-2-associated X protein
Bcl-2: B-cell lymphoma 2
Bim: Bcl-2 interacting mediator of cell death
CaM: calmodulin
CaMKII: Ca2+-calmodulin-dependent protein kinase II
CaMKK: Ca2+-calmodulin-dependent protein kinase kinase
Ca^2+^-ATPase: Ca^2+^-pumping ATPase
DGP: diabetic gastroparesis
EGFP: enhanced green fluorescent protein
eIF4E: eukaryotic translation initiation factor 4E
ELISA: enzyme-linked immunosorbent assay
HBS: Hank buffer salt
HBSS: Hank’s balanced salt solution
HRP: horseradish peroxidase
IP3: inositol trisphosphate
MDM2: murine double-minute 2
mTOR: mammalian target of rapamycin
NC: normal control
PI: propidium iodide
PKA: protein kinase A
PLC: phospholipidol C
p70S6K: p70 S6 ribosomal protein kinase
ROS: reactive oxygen species
STZ: streptozotocin
TSC-1: tuberous sclerosis 1
2-DG: 2-Deoxy-d-glucose
4E-BP1: eukaryotic translation initiation factor 4E binding protein1
α-SMA: α-smooth muscle actin
